# Cancer: A Multifaceted Enemy and the Precision Oncology Response

**DOI:** 10.3390/ijms25115577

**Published:** 2024-05-21

**Authors:** Elena Levantini

**Affiliations:** Institute of Biomedical Technologies, National Research Council (CNR), Area della Ricerca di Pisa, 56124 Pisa, Italy; elena.levantini@itb.cnr.it

Cancer heterogeneity presents a major obstacle in clinical practice that grants tumor cells remarkable levels of resilience, adaptability, and invasiveness. The complexity arises from each tumor having a unique set of genetic alterations, coupled with the exponential capacity of its molecular landscape to evolve under the selective pressure it undergoes during disease progression and in response to treatments. This intrinsic complexity is further amplified by the dynamic nature of the tumor microenvironment (TME), which is an intricate interplay of immune, endothelial stromal, and neural cells [1] whose crosstalk shapes both tumor behavior and treatment outcomes. Understanding the TME’s complexity paves the way for precision medicine, where treatments are finely tuned to a patient’s unique cancer profile, offering a more targeted and effective response to the disease.

In recent decades, oncology has witnessed a paradigm shift towards precision medicine. The papers selected for the Special Issue “Novel Therapeutic Targets in Cancers 2.0” showcase the latest progress in this field. They highlight the TME’s integral role in reinforcing therapeutic interventions and the critical function of immunotherapy in mobilizing a patient’s own immune defenses against cancer. The collection of twelve papers describes the development of innovative therapeutic agents, prognostic markers, and diagnostic tools, all designed to enhance the accuracy and efficacy of patient care.

While redefining tumors as manageable chronic conditions remains a distant goal, with lasting remission still infrequent, each discovery adds a unique piece to the intricate mosaic of knowledge, progressively assembling a detailed picture of cancer precision medicine. This vision drives the continuous quest of researchers dedicated to precision oncology.

## The Latest Research Highlights

1. Chromosomal Instability (CIN) and its clinical implications in Breast Cancer: Camargo-Herrera and colleagues conducted a pilot study investigating the role of CIN and clonal heterogeneity in luminal B breast cancer [2]. Their findings suggest that moderate levels of CIN, coupled with distinct aneuploidy patterns across chromosomes, could correlate with specific clinical outcomes like lympho-vascular invasion. These findings point to the potential of using chromosomal patterns as prognostic indicators for this subtype of breast cancer, pending further validation in larger cohorts. The study emphasizes the need to integrate molecular data with traditional clinicopathological factors to enhance the triad of diagnosis, treatment, and prognosis, offering a more personalized approach to patient care.
2. Connective Tissue Growth Factor (CTGF) as Prognostic Marker in Triple-Negative Breast Cancer (TNBC): Research by Lee et al. identified CTGF as a significant prognostic marker for TNBC [3]. As a key component of the extracellular matrix, CTGF is involved in critical cellular functions such as adhesion, migration, proliferation, angiogenesis, and tissue repair. Their study highlights the influence of kahweol, a diterpene compound present in coffee, on modulating CTGF expression and reducing cell motility. These findings suggest that leveraging the modulation of CTGF may represent a step towards the customization of treatment in TNBC. Further research is essential to unravel the interactions of CTGF with other cellular factors that contribute to tumor growth and metastasis. Understanding these interactions is crucial, as CTGF’s role in TNBC biology may extend from being a prognostic marker to being a potential therapeutic target.
3. Biomarkers for Lymphadenopathy Diagnosis: Lee and coauthors uncovered significant discrepancies between fine needle aspiration cytology (FNAC) and tissue biopsy in diagnosing non-Hodgkin’s lymphoma [4]. Their work underscores the importance of incorporating molecular biomarkers into clinical practice to improve diagnostic accuracy and inform treatment decisions. The authors identified a set of molecular biomarkers to improve diagnosis and treatment planning. These include PMPCB (Peptidase, Mitochondrial Processing Subunit Beta) and ENDOG (Endonuclease G), which play protective roles in maintaining mitochondrial DNA integrity; PRKAR2B (Protein Kinase CAMP-Dependent Type II Regulatory Subunit Beta) and FOS (Fos Proto-Oncogene, AP-1 Transcription Factor Subunit), which regulate cell growth and survival; HDAC10 (Histone Deacetylase 10), which is involved in chromatin remodeling and gene silencing; and HMMR (Hyaluronan-Mediated Motility Receptor), which regulates cell motility and interactions with the extracellular matrix. The authors are in the process of developing a genetic chip that underscores the importance of precision medicine and the potential for advanced technologies to enhance healthcare diagnostics.
4. PARP Inhibition and Immune Response in Myeloproliferative Neoplasms (MPNs): Bermes and colleagues explored the combined effects of olaparib, a PARP inhibitor that disrupts DNA repair, with interferon-alpha (IFNα), which is known for its antitumor immunity [5]. Their research sheds light on the potential of exploiting the synthetic lethality between genetic anomalies in MPN (JAK2V617F mutation and BRCA1 haploinsufficiency) and enhancing the immune response. The efficacy of this approach relies on activating the STING protein, which is crucial for the immune system’s response to DNA damage. This pioneering approach exemplifies the essence of precision medicine, where tailored therapies are designed to exploit specific molecular vulnerabilities, potentially transforming the treatment landscape for patients with MPNs. Further corroboration in animal models and a broader patient cohort are essential to confirm that targeting the STING pathway may enhance therapeutic outcomes.
5. NOTCH1 Inhibition in Acute Leukemias: Fischer et al. introduced CAD204520, a new inhibitor designed to selectively inhibit the NOTCH1 signaling pathway. This compound has demonstrated promising efficacy in preclinical studies for the treatment of aggressive MLL-rearranged leukemia, a subtype notorious for its recalcitrance to conventional chemotherapy regimens [6]. CAD204520 differs from traditional chemotherapy agents like cytarabine by specifically targeting the NOTCH1 receptor, thereby potentially circumventing the systemic toxicities associated with broader-acting chemotherapeutics. The encouraging results observed with CAD204520 in preclinical models support further exploration in clinical settings to refine our therapeutic weapons against acute leukemias.
6. Ubiquitin-specific protease 7 (USP7) Inhibition in Neuroblastoma: The research by Le Clorennec et al. on the potential of the USP7 inhibitor Almac4 is a prime example of tailored medicine, which seeks to customize treatment based on individual genetic profiles and disease characteristics. In neuroblastoma, a cancer that primarily affects children, the effectiveness of Almac4 hinges on the presence of an intact and functional TP53 gene [7]. Almac4 activates the p53 pathway (which is crucial for DNA repair and tumor suppression) and reduces the levels of key proteins involved in cancer progression, such as N-myc and EZH2. The study explores a synergistic strategy along the USP7-MDM2-p53 axis, combining Almac4 with the MDM2 inhibitor Nutlin-3, to enhance treatment efficacy in neuroblastoma, particularly in cases with MYCN amplification. While USP7 inhibitors like Almac4 represent a significant stride forward, as they are efficacious at reduced dosages compared with their predecessors, their impact on normal cellular functions requires further testing to confirm their safety and effectiveness in pediatric oncology. The role of USP7 in cancer extends beyond neuroblastoma, as it is implicated in the etiology, progression, and therapeutic response of various cancers.
7. Interplay of Hepatocyte Odd Protein Shuttling/Trans Membrane Ubiquitin-like containing protein 1 (HOPS/TMUB1) and TP53 Mutations in Cancer Progression: The research conducted by Di-Iacovo and coauthors marks a pivotal contribution in the elucidation of the molecular mechanisms supporting cancer pathogenesis, focusing on the interactions between the nucleo-cytoplasmic shuttling protein HOPS/TMUB1 and mutations within the TP53 gene in breast, pancreatic, and lung cancer [8]. The findings reveal that the pathogenicity of p53 mutations goes beyond individual amino acid alterations; it is the coming together of multiple factors that leads to dysregulation of the p53 pathway. Within this framework, HOPS/TMUB1 emerges as a key modulator capable of extending the half-life of p53, thus enhancing its capacity to suppress tumorigenesis. The affinity of HOPS/TMUB1 for variously mutated forms of p53 underscores its role as a critical regulator in the apoptotic process. This is corroborated by patient data indicating a robust correlation between heightened HOPS/TMUB1 expression and apoptosis, a relationship that strengthens with increasing levels of HOPS/TMUB1. The impact of HOPS/TMUB1 on p53 mutants varies, influencing the balance between cell death and survival in different cellular contexts. This is compelling in the context of precision medicine, as the authors reveal how the modulation of HOPS/TMUB1 expression can influence the apoptotic balance in cancer cells. For instance, in H1975 lung cancer cells, HOPS/TMUB1 may increase MYC expression, which could lead to uncontrolled cellular proliferation, yet it retains the ability to trigger apoptosis. In contrast, SKBR3 breast cancer cells with HOPS/TMUB1 overexpression do not exhibit MYC-induced growth but do show an increase in TP63 expression, which supports apoptosis even in the presence of mutated p53. The overexpression of HOPS/TMUB1, particularly in cells with common p53 mutations, induces apoptosis as in the cellular response to DNA damage, highlighting its sentinel role in determining cellular destiny. Ultimately, this research sheds light on the complex interplay between TP53 mutations and HOPS/TMUB1 and their consequential impact on cancer progression. The exploration continues as we delve deeper into these interactions in pursuit of novel treatments for the fight against cancer.
8. Radiotherapy and Immunotherapy Synergy: Luna-Gutierrez et al. introduced a novel treatment that combines a targeted radiotherapy approach with immunotherapy’s systemic defense [9]. This innovative dual therapy employs PD-L1 inhibitory peptides, labeled with radioactive isotopes, to administer precise ablative radiation doses directly within the TME while preserving the integrity of surrounding healthy tissue. Demonstrating significant uptake in lung micro-metastases, rapid clearance from the bloodstream, and minimal impact on healthy tissues, this method promises to stand at the forefront of innovative cancer therapies. Given the almost ubiquitous expression of PD-L1 in virtually all tumors, such therapeutic applications may expand well beyond the lung cancer field. The activation of the abscopal effect, named for the Latin word “ab”, meaning “away from”, and the Greek “skopós”, meaning “aim/target”, which may trigger a systemic antitumor reaction against distant cancer cells, is noteworthy. This research hints at a future where cancer cells can no longer elude treatments. Additional preclinical testing is, however, required to validate the effectiveness of this treatment methodology.
9. Annexin A5 in Drug Delivery: Jing and colleagues provided an in-depth exploration of human annexin A5 (hAnxA5), discussing its role in cellular processes and its potential in medical applications, particularly in targeted drug delivery [10]. Originally identified as an anticoagulant, hAnxA5 has since been implicated in a variety of biological functions, including membrane transport, the regulation of ion channels, and the induction of apoptosis. Its influence extends to human physiology and pathology, influencing coagulation, cardiovascular health, autoimmune conditions, cancer progression, and more. The protein’s ability to bind to phosphatidylserine (PS) on the surface of apoptotic cells makes it a valuable ally for the direct administration of therapeutic agents into cancer cells. This could potentially improve the efficacy of chemotherapeutic treatments while concurrently minimizing side effects. The versatility of hAnxA5 does not end there; its potential as a biomarker for a spectrum of conditions, including cancer, cardiac failure, renal damage, and respiratory illnesses such as asthma, opens up possibilities for its use in diagnostics. In addition, hAnxA5 could serve as a noninvasive imaging tool when tagged with isotopes, detect apoptosis as an early sign of disease, and serve as a personalized diagnostic tool. However, the authors caution that the road ahead is still uncertain, and that a concerted research effort is necessary to unlock the full potential of hAnxA5 in advancing biomedical technologies and enhancing both therapeutic and diagnostic applications.
10. Advancements in Targeted Therapies: Choi et al. examined advancements in targeted therapies for lung, colorectal, and prostate cancers, detailing the discovery of molecular targets and the development of FDA-approved drugs and emphasizing the recent shift towards personalized medicine [11]. Their review outlines the transition from conventional surgical interventions to precision drugs like osimertinib and crizotinib for lung cancer, particularly NSCLC, which has seen significant strides in targeting EGFR and ALK mutations. The study also touches on progress in addressing MET, ROS1, RET, and BRAF V600E alterations. Then, they turn their attention to colorectal cancer, noting its rising incidence among younger demographics. They discuss the advancements in targeted therapies, particularly VEGF and EGFR pathway inhibitors like bevacizumab and cetuximab, which are especially effective in metastatic cases. The promise of combination therapies targeting BRAF V600E mutations and the exploration of TGF-β inhibitors in preventing metastasis is discussed, together with the FDA’s accelerated approval of treatments targeting HER2 and the potential of immune checkpoint inhibitors in tumors with a high mutational burden. In the context of prostate cancer, the second leading cause of cancer-related deaths, whose incidence is on the rise, the evolution of antiandrogen treatments, including enzalutamide and apalutamide, is examined, and the FDA’s approval of darolutamide, enhancing metastasis-free survival, is also highlighted. The review acknowledges the efficacy of abiraterone acetate and PARP inhibitors for genetically predisposed patients and points to ongoing research on PSMA (prostate-specific membrane antigen) for radioligand therapy, angiogenesis inhibition, and immunotherapy. The authors emphasize the evolving landscape of cancer research, advocating for a focus on overcoming the development of resistance, identifying biomarkers, and developing optimal combinations as treatment strategies. This comprehensive review reflects the dynamic and progressive nature of research on targeted cancer therapies.
11. Forkhead box protein M1 in the pathogenesis of Malignant Peripheral Nerve Sheath Tumors (MPNTSs): Voigt and colleagues highlighted the significance of FOXM1 in the pathogenesis of MPNSTs, a type of aggressive sarcoma that arise in patients with Neurofibromatosis Type I, which inherently increases the susceptibility to such tumors [12]. The research points to the need for a deeper understanding of the molecular and genetic alterations that catalyze the transformation from benign neurofibromas to MPNSTs. They present FOXM1, a central regulator in the cell cycle whose overexpression correlates with disease progression, as a promising target for therapeutic intervention. The study shows that FOXM1’s expression is modulated by ARF, MEK-ERK1/2, and p53, which is itself associated with adverse patient prognoses. The interaction of FOXM1 with other molecular players like RB1 and EZH2 impacts cellular proliferation and resistance to oncological treatments. The authors suggest that exploring the regulation of FOXM1 and the pathways frequently altered in MPNSTs, particularly MEK and CDK4/6, could lead to more effective treatments for MPNST. This approach is reflective of precision medicine’s goal to develop targeted interventions based on a deep understanding of the disease at a molecular level.
12. New Treatments for Urothelial Carcinoma: Clasp et al. reviewed the current challenges in the treatment of urothelial carcinoma, emphasizing the urgency for new therapeutic approaches tailored to patient-specific conditions, particularly for patients with high-risk non-muscle-invasive bladder carcinoma (NMIBC) who exhibit resistance to standard Bacillus Calmette–Guérin (BCG) therapy [13]. The review reports the promise of innovative immunotherapies targeting TIGIT (T cell immunoreceptor with Ig and ITIM domains) and LAG-3 (lymphocyte activation gene-3), which may enhance the efficacy of pembrolizumab. Ongoing studies are assessing their safety and therapeutic potential. Emerging treatments like Erdafitinib, Tislelizumab, and Enfortumab vedotin demonstrate efficacy against BCG-refractory bladder cancer. Erdafitinib targets FGFR alterations, Tislelizumab extends bladder conservation, and Enfortumab vedotin improves survival rates in advanced stages. Novel therapeutics are being developed, such as HX008, an anti-PD-1 humanized monoclonal antibody, and EG-70, a plasmid encoding immune activators,; both are undergoing clinical trials. As new therapies emerge, there is a pressing need for selective biomarkers to guide treatment options and clinical trial design given the disease’s heterogeneity. The review concludes by advocating for the exploration of bladder-conserving strategies and novel molecular targets for therapy. It discusses the necessity for alternatives to radical cystectomy, considering the profound impact of such surgeries on patients’ quality of life.

As we draw this Editorial to a close, we reflect on the quest for knowledge as a collaborative endeavor, aiming to translate preclinical discoveries into effective diagnostic and therapeutic strategies. Each milestone paves the way toward a deeper understanding of cancer biology, envisioning a future in which cancer becomes a manageable condition through the application of molecular precision oncology. The ongoing series “Novel Therapeutic Targets in Cancers” invites contributions to its third edition, continuing the shared journey of discovery and innovation in cancer treatment. Together, we will continue to unravel the complexities of cancer and design patient-tailored treatments that not only extend life but also enhance its quality.
